# Anticoagulação Crônica em Pacientes com Fibrilação Atrial e COVID-19: Uma Revisão Sistemática e Metanálise

**DOI:** 10.36660/abc.20230470

**Published:** 2024-03-21

**Authors:** Isabela Landsteiner, Jonathan A. Pinheiro, Nicole Felix, Douglas Mesadri Gewehr, Rhanderson Cardoso

**Affiliations:** 1 Massachusetts General Hospital Boston Massachusetts EUA Massachusetts General Hospital, Boston, Massachusetts – EUA; 2 Universidade de Fortaleza Fortaleza CE Brasil Universidade de Fortaleza, Fortaleza, CE – Brasil; 3 Universidade Federal de Campina Grande Campina Grande PB Brasil Universidade Federal de Campina Grande, Campina Grande, PB – Brasil; 4 Instituto do Coração de Curitiba Curitiba PR Brasil Instituto do Coração de Curitiba, Curitiba, PR, Brasil; 5 Brigham and Women's Hospital and Harvard Medical School Boston Boston Massachusetts EUA Brigham and Women's Hospital and Harvard Medical School, Boston, Massachusetts – EUA

**Keywords:** Anticoagulantes, Fibrilação Atrial, COVID-19, Inibidores do Fator Xa, Vitamina K

## Abstract

**Fundamento:**

A doença por coronavírus 2019 (COVID-19) está associada à hipercoagulabilidade. Permanece incerto se a anticoagulação contínua para fibrilação atrial (FA) em pacientes que posteriormente contraem COVID-19 melhora os desfechos clínicos.

**Objetivos:**

Comparar a anticoagulação oral crônica com ausência de anticoagulação prévia em pacientes com FA que contraíram uma infecção por COVID-19 em relação aos desfechos de mortalidade por todas as causas, mortalidade por COVID-19, admissão em unidade de terapia intensiva (UTI) e hospitalização.

**Métodos:**

Buscamos sistematicamente no PubMed, Embase e Cochrane Library estudos elegíveis desde o início até dezembro de 2022. Incluímos estudos que compararam desfechos de COVID-19 em pacientes com e sem anticoagulação crônica prévia para FA. Foram agrupadas razões de risco (RR) com intervalos de confiança (IC) de 95% por meio de um modelo de efeitos aleatórios. O nível de significância foi estabelecido em p < 0,05. As avaliações da qualidade e do risco de viés foram realizadas de acordo com as recomendações da Cochrane.

**Resultados:**

Foram identificados 10 estudos abrangendo 1.177.858 pacientes com COVID-19 e FA, dos quais 893.772 (75,9%) estavam em anticoagulação crônica prévia para FA. Em pacientes com COVID-19, a anticoagulação crônica para FA reduziu significativamente a mortalidade por todas as causas (RR 0,75; IC 95% 0,57 a 0,99; p = 0,048; I^2^ = 89%) e a mortalidade relacionada à COVID-19 (RR 0,76; IC 95% 0,72 a 0,79; p < 0,001; I^2^ = 0%) quando comparada com a ausência de anticoagulação prévia. Em contrapartida, não houve diferença entre os grupos em relação à hospitalização (RR 1,08; IC 95% 0,82 a 1,41; p = 0,587; I^2^ = 95%) ou internação em UTI (RR 0,86; IC 95% 0,68 a 1,09; p = 0,216; I^2^ = 69%).

**Conclusões:**

Nesta metanálise, a anticoagulação crônica para pacientes com FA que contraíram COVID-19 foi associada a taxas significativamente mais baixas de mortalidade por todas as causas e mortalidade relacionada à COVID-19 em comparação com a ausência de anticoagulação anterior.

**Figure:**
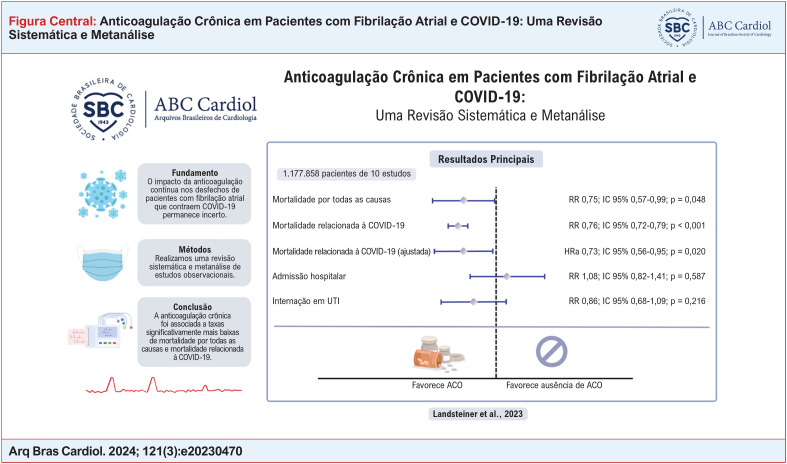


## Introdução

A doença por coronavírus 2019 (COVID-19), causada pelo coronavírus 2 da síndrome respiratória aguda grave (SARS-CoV-2), surgiu em dezembro de 2019, levando a uma pandemia global com uma elevada carga de morbidade, mortalidade e dificuldades econômicas.^1^ A Organização Mundial da Saúde relatou um total de 767 milhões de casos confirmados e 6,9 milhões de mortes em todo o mundo até 5 de julho de 2023.^2^

A infecção por SARS-CoV-2 está associada a uma grande variedade de apresentações clínicas não respiratórias, incluindo trombose microvascular pulmonar e coagulação anormal, mesmo em casos leves. A fisiopatologia por trás disso refere-se principalmente à hipercoagulabilidade imune-induzida secundária à resposta do paciente à infecção.^3,4^ Levando isso em consideração, as diretrizes recomendam que os pacientes com COVID-19 em uso de anticoagulação oral para condições subjacentes e sem quaisquer contraindicações com relação a ela não interrompam esses tratamentos.^5^

A terapia prolongada com anticoagulante oral (ACO) é uma medida essencial para evitar eventos tromboembólicos em pacientes com fibrilação atrial (FA). A hipercoagulabilidade associada à infecção por SARS-CoV-2 levanta preocupações sobre como a anticoagulação contínua em pacientes com FA que posteriormente contraem COVID-19 pode afetar os desfechos clínicos.^5^ Portanto, realizamos uma revisão sistemática e metanálise comparando os desfechos da anticoagulação versus a ausência de anticoagulação em pacientes com FA que desenvolveram COVID-19.

## Métodos

Esta revisão sistemática e metanálise foram realizadas seguindo as diretrizes da Manual da Colaboração Cochrane para Revisões Sistemáticas de Intervenções e da declaração dos Principais Itens para Relatar Revisões Sistemáticas e Metanálises (PRISMA).^6,7^ A metanálise foi registrada prospectivamente no Registro Prospectivo de Revisões Sistemáticas (PROSPERO) sob o número de protocolo CRD42022341926.

### Estratégia de busca e extração de dados

Buscamos sistematicamente no PubMed, Embase e na Biblioteca Cochrane desde o início até dezembro de 2022. Depois de remover as duplicatas, dois autores (I.L. e N.F.) selecionaram títulos e resumos e avaliaram independentemente os artigos de texto integral para inclusão com base em critérios pré-especificados. As discrepâncias foram resolvidas por consenso entre os autores. Além disso, utilizamos a técnica de *backward snowballing* (isto é, revisão de referências) para identificar textos relevantes de artigos identificados na pesquisa original.

Nossa estratégia de pesquisa incluiu os seguintes descritores em ciências da saúde: "atrial fibrillation", "oral anticoagulant", "OACs", "NOAC", "non-vitamin K", "novel anticoagulant", "DOAC", "DOACs", "direct oral anticoagulant", "dabigatran", "apixaban", "edoxaban", "rivaroxaban", "VKA", "vitamin K antagonist", "warfarin", "LMWH", "low-molecular-weight heparin", "low molecular weight heparin", "enoxaparin", "bivalirudin", "dalteparin", "fondaparinux", "COVID-19", "coronavirus disease 19", "coronavirus disease-19", "SARS-CoV-2."

### Critérios de elegibilidade

Esta revisão sistemática e metanálise incluíram estudos que (1) eram relatórios completos publicados em jornais indexados ou resumos de grandes conferências científicas; (2) incluíram pacientes adultos (idade ≥ 18 anos) previamente diagnosticados com FA que posteriormente contraíram COVID-19 confirmada por teste validado; (3) estratificaram pacientes em uso ou não de anticoagulação crônica para FA; (4) relataram qualquer um dos nossos resultados de interesse, nomeadamente: mortalidade por todas as causas, mortalidade por COVID-19, internação em unidade de terapia intensiva (UTI) e hospitalização; e (5) foram publicados em inglês ou espanhol. Excluímos (1) estudos com populações sobrepostas; (2) relatos de casos, séries de casos, cartas ao editor, comentários ou editoriais; e (3) estudos sem grupo controle.

### Avaliação de qualidade

Os estudos observacionais foram avaliados usando o risco de viés de estudos de intervenção não randomizados (Risk of Bias Summary for Non-randomized Studies – ROBINS-I) para avaliar a qualidade metodológica dos estudos incluídos, uma ferramenta baseada em respostas às questões de sinalização e análises para cada domínio de viés e para risco geral de viés, o que permite classificar cada estudo como risco de viés "baixo", "moderado", "grave" ou "crítico".^8^ O efeito de estudos pequenos (viés de publicação) foi avaliado com gráficos de funil e teste de regressão de Egger.^9^

### Análise estatística

Os desfechos binários foram resumidos usando o modelo de efeitos aleatórios de Mantel-Haenszel (MH), com risco relativo (RR) e intervalo de confiança (IC) de 95% como medida do tamanho do efeito. Utilizamos o estimador Sidik-Jonkman (método de variância do erro do modelo) para calcular a variância da heterogeneidade τ^2^, uma vez que se esperava que o grau de heterogeneidade fosse substancial.^10^

O *hazard ratio* ajustado (HRa) para múltiplos fatores de confusão, se relatados, foi agrupado usando o modelo de efeitos aleatórios de MH com IC de 95% e estimador de Paule-Mandel para cálculo de variância de heterogeneidade. A heterogeneidade entre os estudos foi avaliada com a estatística Q de Cochrane (e o qui-quadrado resultante), com p ≤ 0,10 considerado estatisticamente significativo. O teste estatístico I^2^ de Higgins e Thompson foi usado para medir a consistência.^11^ Um valor de 0% indica nenhuma heterogeneidade observada, e valores de 1% a 25%, 26% a 50% e > 50% indicam heterogeneidade baixa, moderada e substancial, respectivamente.^11^ A significância estatística foi estabelecida em p < 0,05 e todos os testes foram bicaudais. Realizamos todos os cálculos e gráficos com o software R versão 4.2.2 (R Core Team 2021) usando os pacotes de extensão "meta", "metafor" e "dmetar".^12-15^

### Abordando a heterogeneidade

Realizamos uma análise de exibição gráfica de heterogeneidade (GOSH) para mortalidade por todas as causas para identificar possíveis *outliers* e estudos influentes.^16^ Primeiro, foi gerado um gráfico GOSH. Em seguida, foram aplicados 3 algoritmos de aprendizado de máquina (AM) não supervisionados para detectar *clusters* nos dados do gráfico GOSH, da maneira seguinte: (1) o algoritmo *k*-means;^17^ (2) agrupamento espacial baseado em densidade de aplicações com ruído (DBSCAN);^18^ e (3) modelos de mistura gaussiana.^19^

Alternativamente, aplicamos as funções "find.outliers" e "InfluenceAnalysis" (pacote R dmetar) para mortalidade por todas as causas, hospitalização e internação em UTI para auxiliar na identificação de possíveis *outliers*. A função "find.outliers" define o estudo como um *outlier* se o intervalo de confiança do estudo não se sobrepuser ao intervalo de confiança do efeito agrupado. A função "InfluenceAnalysis", por sua vez, gera 3 gráficos de diagnóstico: (1) o gráfico de Baujat, usado para identificar estudos que tiveram altas contribuições para a heterogeneidade nos dados meta-analíticos;^20^ (2) a análise de sensibilidade de exclusão, removendo iterativamente um estudo de cada vez para garantir que os resultados não dependessem de um único estudo; e (3) diagnósticos de influência segundo Viechbauer e Cheung.^21^

## Resultados

### Seleção dos estudos e características de linha de base

**Figura 1:**
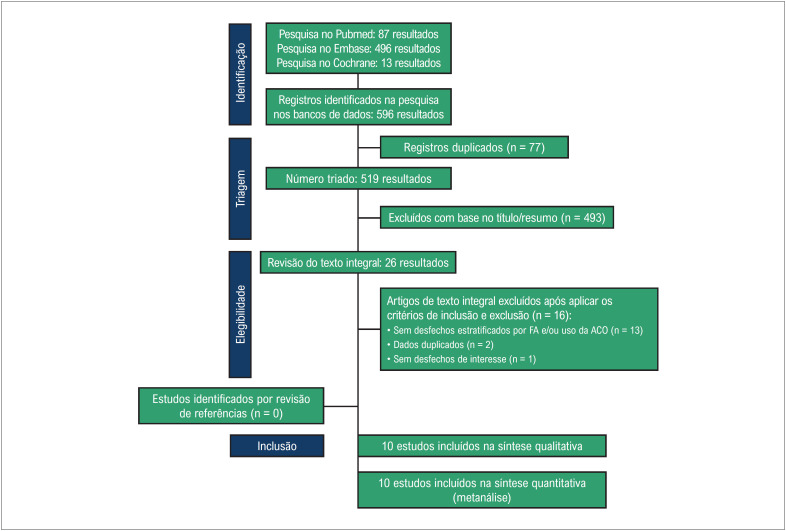
Fluxograma PRISMA de triagem e seleção de estudos. ACO: anticoagulante oral; FA: fibrilação atrial.

Conforme ilustrado na Figura 1, foram identificados 596 estudos, dos quais 493 foram excluídos com base na revisão do título ou do resumo. Foram completamente revisados 26 estudos, com base em nossos critérios de inclusão. Após a avaliação final, restaram 10 manuscritos elegíveis para inclusão nesta metanálise.^22-31^ Foram incluídos 1.177.858 pacientes, dos quais 893.772 (75,9%) estavam em uso de anticoagulantes para FA antes da infecção por COVID-19. A média de idade variou de 71 a 82 anos e 629.628 pacientes eram do sexo masculino (55,5%). As características individuais do estudo são relatadas na Tabela 1.

**Tabela 1:** Características de linha de base dos estudos incluídos

Estudo	Número de pacientes ACO / sem ACO	Sexo masculino, n (%)	Idade média, (anos)	HTN, n (%)	IC, n (%)	AVC, AIT ou embolia sistêmica, n (%)	Doença renal, n (%)	Doença hepática, n (%)	DAP, n (%)	DM, n (%)	Doença pulmonar, n (%)
Ageno, 2021^22^	43 / 111	91 (59,1)	81,0†	104 (67,5)	52 (33,8)	24 (15,6)	NA	NA	NA	30 (19,4)	43 (27,9)
Denas, 2021^23^	559 / 559	608 (54,4)	NA	972 (86,9)	184 (16,4)	169 (15,1)	96 (8,6)	11 (1,0)	27 (2,4)	298 (24,0)	NA
Flam, 2020^24^	103.703 / 36.875	87.508 (62,2)	73,6†	NA	30.986 (22,0)	28.803 (20,5)	7.823 (5,6)	2.227 (1,6)	NA	NA	27.855 (19,8)
Fumagalli, 2022^25^	91 / 85	91 (51,7)	82,0	125 (71,0)	55 (31,0)	23 (13,0)	36 (20,0)	3 (1,7)	37 (21,0)	58 (32,0)	37 (21,0)
Gómez, 2022^26^	1.361 / 392	1.065 (59,1)	78,1*	1.430 (79,4)	568 (31,5)	NA	NA	NA	174 (9,6)	555 (30,8)	269 (14,9)
Handy, 2022^27^	722.737 / 187.133	480.676 (52,8)	79,0	630.245 (69,2)	225.746 (24,8)	180.958 (19,9)	293.949 (32,3)	7.983 (0,9)	137.666 (15,1)	245.195 (26,9)	NA
Louis, 2022^28^	361 / 269	354 (56,1)	77,4	542 (86,0)	310 (49,2)	92 (14,6)	229 (36,3)	57 (9,0)	7 (1,1)	280 (44,4)	83 (13,2)
Rivera-Caravaca, 2022^29^	675 / 7.023	4.500 (58,4)	63,0†	3.718 (48,3)	174 (2,3)	570 (7,4)	484 (6,3)	271 (3,5)	645 (8,4)	1.455 (18,9)	523 (6,8)
Wong, 2022^30^	52.832 / 18.271	54.735 (76,9)	71,0†	29.683 (41,7)	7.124 (10,0)	2.244 (3,1)	10.524 (14,8)	NA	634 (0,9)	9.190 (12,9)	6.815 (9,6)
Zadeh, 2022^31^	11.410 / 33.368	NA	NA	NA	44.778 (100,0)	NA	NA	NA	NA	NA	NA

* Dados referentes a pacientes não falecidos.

† Dados referentes a pacientes anticoagulados. ACO: anticoagulante oral; AIT: ataque isquêmico transitório; AVC: acidente vascular cerebral; DAP: doença arterial periférica; DM: diabetes mellitus; HTN: hipertensão; IC: insuficiência cardíaca.

Dada a natureza não randomizada dos estudos, relatamos características basais estratificadas por uso versus não uso de anticoagulação entre os 7 estudos que relataram esses dados (Tabela 2). Como esperado, houve uma carga maior de comorbidades em pacientes com FA em uso de ACO em comparação com aqueles não em uso de ACO, incluindo hipertensão, insuficiência cardíaca, doença renal e eventos tromboembólicos prévios. Os pacientes em uso de ACO também eram mais velhos do que aqueles que não estavam anticoagulados.

**Tabela 2:** Características dos estudos individuais subdivididas por uso de anticoagulação

Estudo	Número de pacientes ACO / sem ACO	Sexo masculino, n (%) ACO / sem ACO	Idade média, (anos) ACO / sem ACO	HTN, n (%) ACO / sem ACO	IC, n (%) ACO / sem ACO	AVC, AIT ou embolia sistêmica, n (%) ACO / sem ACO	Doença renal, n (%) ACO / sem ACO	Doença hepática, n (%) ACO / sem ACO	DAP, n (%) ACO / sem ACO	DM, n (%) ACO / sem ACO	Doença pulmonar, n (%) ACO / sem ACO
Ageno, 2021^22^	43 / 111	23 (53,5) / 68 (61,3)	81,0 / 80,0	30 (69,8) / 74 (66,7)	24 (55,8) / 28 (25,2)	8 (18,6) / 16 (14,4)	NA	NA	NA	10 (23,2) / 20 (18,0)	11 (25,6) / 32 (28,8)
Denas, 2021^23^	559 / 559	303 (54,2) / 306 (54,7)	NA	490 (87,7) / 485 (86,8)	93 (16,6) / 91 (16,3)	78 (14,0) / 91 (16,3)	47 (8,4) / 49 (8,8)	6 (1,1) / 5 (0,9)	15 (2,7) / 12 (2,1)	132 (23,6) / 137 (24,5)	NA
Flam, 2020^24^	103.703 / 36.875	62.488 (60,3) / 25.020 (67,9)	73,6 / 66,4	NA	26.544 (25,6) / 4.442 (12)	17.650 (17) / 2.853 (7,7)	6.082 (5,9) / 1.741 (4,7)	1.445 (1,4) / 832 (2,3)	NA	NA	21.762 (21) / 6.093 (16,5)
Handy, 2022^27^	722.737 / 187.133	384.260 (53,2) / 96.416 (10,6)	79,0 / 79,0	600.623 (83,1) / 124.731 (66,6)	228.877 (31,6) / 33.723 (18,0)	183.140 (25,3) / 30.370 (16,2)	284.379 (39,3) / 55.984 (29,9)	8.462 (1,17) / 2.543 (1,40)	159.892 (22,1) / 33.720 (18,0)	242.060 (33,5) / 47.979 (25,6)	NA
Louis, 2022^28^	361 / 269	206 (57,1) / 148 (55)	78,5 / 75,9	311 (86,1) / 231 (85,9)	200 (55,4) / 110 (40,9)	62 (17,2) / 30 (11,2)	113 (31,3) / 65 (24,2)	26 (7,2) / 31 (11,5)	4 (1,1) / 3 (1,1)	158 (43,8) / 122 (45,4)	55 (15,2) / 28 (10,4)
Rivera-Caravaca, 2022^29^	675 / 7.023	403 (59,7) / 4.097 (58,3)	80,0 / 63,0	543 (80,3) / 3.176 (45,2)	46 (6,8) / 128 (1,8)	131 (19,4) / 439 (6,3)	115 (17) / 369 (5,3)	33 (4,9) / 238 (3,4)	102 (15,1) / 543 (7,7)	198 (29,3) / 1.257 (17,9)	104 (15,4) / 419 (6,0)
Wong, 2022^30^	52.832 / 18.271	41.870 (79,2) / 12.865 (70,4)	71,0 / 69,0	22.061 (41,8) / 7.622 (41,7)	5.700 (10,8) / 1.424 (7,8)	1.041 (2,0) / 336 (1,8)	8.477 (16,0) / 2.047 (11,2)	NA	426 (0,8) / 208 (1,1)	6.508 (12,3) / 2.682 (14,6)	7.372 (14) / 2.302 (12,6)

ACO: anticoagulante oral; AIT: ataque isquêmico transitório; AVC: acidente vascular cerebral; DAP: doença arterial periférica; DM: diabetes mellitus; HTN: hipertensão; IC: insuficiência cardíaca.

### Análise agrupada de todos os estudos

**Figura 2:**
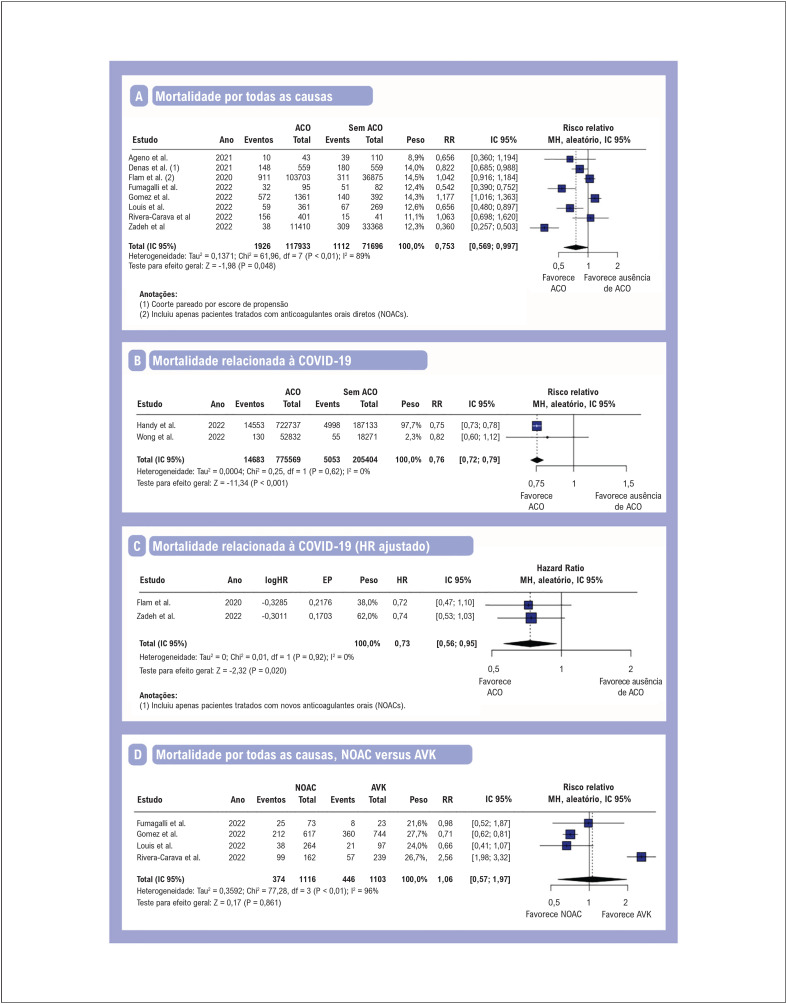
Metanálise de desfechos de mortalidade em pacientes com fibrilação atrial em terapia com ACO. Gráficos de floresta apresentando risco relativo (RR) ou hazard ratio (HR) e intervalo de confiança de 95% (IC) para (A) mortalidade por todas as causas, (B) mortalidade relacionada à COVID-19, (C) mortalidade relacionada à COVID-19 (HR ajustado) e (D) mortalidade por todas as causas com NOAC versus AVK. ACO: anticoagulantes orais; AVK: antagonista da vitamina K; EP: erro padrão; HR: hazard ratio; IC: intervalo de confiança; MH: Mantel-Haenszel; NOAC: anticoagulante oral não-antagonista da vitamina K; RR: risco relativo.

Os desfechos de mortalidade estão resumidos na Figura 2A-D. A mortalidade por todas as causas (Figura 2A) e a mortalidade relacionada à COVID-19 (Figura 2B) foram significativamente reduzidas entre os pacientes que receberam terapia com ACO em comparação com aqueles sem uso prévio de ACO. Realizamos uma análise de subgrupo pré-especificada agrupando estudos que relataram HRa para mortalidade por COVID-19, levando em consideração vários fatores de confusão. Na análise de sensibilidade, a terapia com ACO permaneceu significativamente associada à redução da mortalidade relacionada à COVID-19 (Figura 2C). Quando estratificado pelo tipo de terapia com ACO, não houve diferença significativa entre a terapia com anticoagulantes oral não-antagonistas da vitamina K (NOAC) versus a terapia com antagonistas da vitamina K (AVK) para mortalidade por todas as causas (Figura 2D).

**Figura 3:**
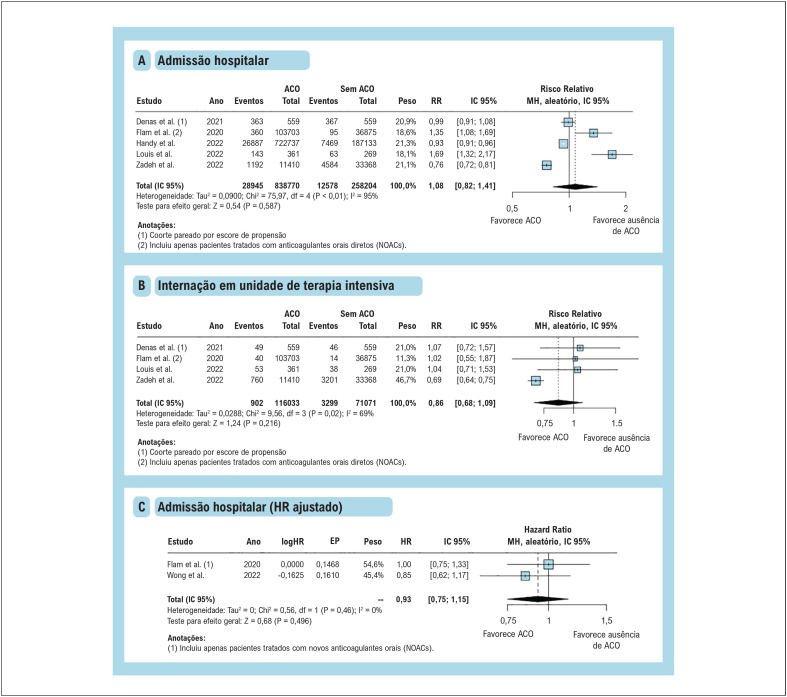
Metanálise de desfechos hospitalares em pacientes com fibrilação atrial em terapia com ACO. Gráficos de floresta apresentando risco relativo (RR) ou hazard ratio (HR) e intervalo de confiança de 95% (IC) para (A) admissão hospitalar, (B) internação em unidade de terapia intensiva e (C) admissão hospitalar (HR ajustado). ACO: anticoagulantes orais; HR: hazard ratio; IC: intervalo de confiança; MH: Mantel-Haenszel; RR: risco relativo.

As incidências de hospitalização (Figura 3A) e internação em UTI (Figura 3B) foram semelhantes entre a terapia com ACO versus sem ACO, com heterogeneidade substancial entre os estudos em ambos os resultados. Considerando isso, realizamos uma análise de sensibilidade para o desfecho de hospitalização, incluindo apenas estudos que relataram HRa por modelos multivariados ou pareamento por escore de propensão, que obteve resultados consistentes com a análise geral e eliminou a heterogeneidade entre os estudos (Figura 3C).

### Abordando a heterogeneidade

**Figura 4:**
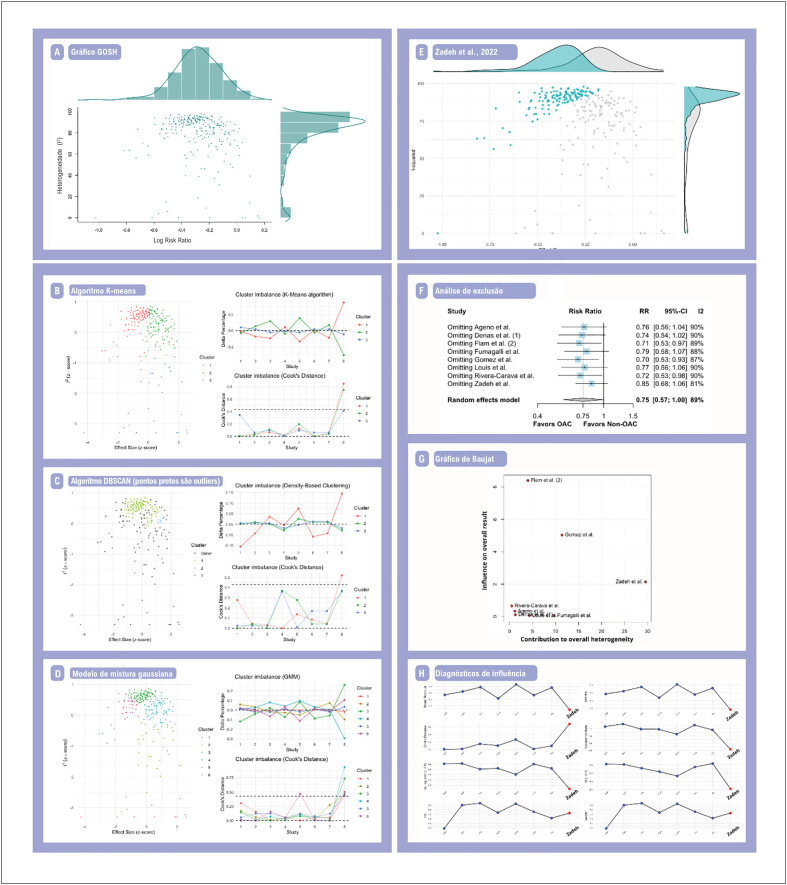
Abordando a heterogeneidade. (4A) Gráfico GOSH; (4B) Algoritmo K-means; (4C) Algoritmo DBSCAN; (4D) Modelo de mistura gaussiana; (4E) Gráfico GOSH com o subconjunto correspondente (Zadeh et al., 2022), incluindo os potenciais outliers coloridos em ciano; (4F) Análise de exclusão (leave-one-out); (4G) Gráfico de Baujat; (4H) Diagnósticos de influência. ACO: anticoagulantes orais; DBSCAN: agrupamento espacial baseado em densidade de aplicações com ruído; GMM: modelo de mistura gaussiana; GOSH: exibição gráfica de heterogeneidade; HR: hazard ratio; IC: intervalo de confiança; MH: Mantel-Haenszel; RR: risco relativo.

Considerando a heterogeneidade significativa entre estudos obtida nos principais desfechos, realizamos análises GOSH para mortalidade por todas as causas, nosso desfecho primário. O gráfico GOSH ilustra o tamanho do efeito plotado em relação ao I^2^ para todas as combinações possíveis de estudos. Os 255 subconjuntos possíveis de metanálise (2κ – 1 combinações possíveis) para mortalidade por todas as causas são apresentados como um gráfico GOSH na Figura 4A. Ao analisar o padrão dos nossos dados, descobrimos que a maioria dos valores está concentrada em um *cluster* com elevada heterogeneidade. A distribuição de I^2^ é relativamente unimodal, embora pareça haver algumas combinações de estudos esparsamente distribuídas para as quais a heterogeneidade estimada é levemente inferior, com um tamanho de efeito agrupado razoavelmente preservado, resultando em uma distribuição global de I^2^ distorcida para baixo e para a esquerda.

Para identificar quais estudos podem ter causado esse formato, aplicamos 3 algoritmos de AM não supervisionados, detalhados nos Métodos, para detectar *clusters* nos dados do gráfico GOSH (Figuras 4B-D). Em última análise, um potencial *outlier* foi identificado pelas ferramentas de AM.^31^ O subconjunto correspondente, incluindo o nosso potencial *outlier*, é demonstrado na Figura 4E. Em resumo, a análise GOSH mostrou que a heterogeneidade não mudou significativamente, independentemente da publicação que foi excluída; o efeito geral também não mudou significativamente. Isso é mais consistente com a interpretação de que nossos resultados são robustos e confiáveis, embora a heterogeneidade geral entre os estudos possa ser significativa.

Como a análise GOSH permaneceu heterogênea para mortalidade por todas as causas, exploramos ainda mais a influência de cada estudo realizando uma análise de sensibilidade de exclusão (Figura 4F), traçando o gráfico de Baujat (Figura 4G) e realizando diagnósticos de influência (Figura 4H). A análise de exclusão e o gráfico de Baujat mostraram que Zadeh et al. teve a maior contribuição para a alta heterogeneidade, consistente com os resultados da análise GOSH.^31^ Além disso, as estimativas de efeito agrupado (RR) na análise de exclusão variaram de 0,70 a 0,85. Ao excluir Flam et al.,^24^ Gómez et al.^26^ e Rivera-Caravaca et al.,^29^ o tamanho do efeito resultante permaneceu estatisticamente significativo. Paralelamente, aplicamos a função InfluenceAnalysis (pacote R dmetar) para verificar se outra abordagem para reconhecer casos influentes detectou os mesmos *outliers* encontrados nas análises mencionadas. Diagnósticos de influência caracterizaram quais estudos se enquadram bem em nosso modelo de metanálise e quais não.

### Avaliação de qualidade

A ferramenta ROBINS-I identificou 7 estudos com risco geral moderado de viés, enquanto 3 foram identificados como apresentando risco geral grave de viés (Tabela Suplementar S1). Os gráficos de funil para mortalidade por todas as causas, hospitalização e internação na UTI foram levemente assimétricos (Tabela Suplementar S2A-C). No entanto, o teste de Egger para viés de publicação foi estatisticamente significativo apenas para o desfecho de internação em UTI (p = 0,04) (Tabela Suplementar S2C).

## Discussão

Nesta revisão sistemática e metanálise de 10 estudos observacionais e 1.177.858 pacientes comparando a anticoagulação oral crônica com ausência de anticoagulação prévia em pacientes com FA que contraíram COVID-19, nossos principais achados foram os seguintes: (1) a mortalidade por todas as causas e a mortalidade relacionada à COVID-19 foram significativamente menores em pacientes em terapia crônica com ACO; e (2) a associação da terapia com ACO a uma redução da mortalidade relacionada à COVID-19 persistiu mesmo após uma análise conjunta de *hazard ratios* ajustados para múltiplos fatores de confusão.

A associação entre FA e desfechos adversos está bem documentada na literatura, uma vez que a FA aumenta significativamente o risco de acidente vascular cerebral, embolia sistêmica e mortalidade.^32-35^ Neste contexto, a anticoagulação oral melhora substancialmente os desfechos cardiovasculares e, em última análise, a sobrevivência em pacientes com AF.^32-34^ Especificamente, foi demonstrado que a anticoagulação oral reduz o risco de acidente vascular cerebral isquêmico em 64% e a mortalidade por todas as causas em 26%.^34^ Como a FA está associada a piores desfechos em pacientes com COVID-19, o benefício da anticoagulação pode ser ainda maior em pacientes com FA infectados por COVID-19.^36^ Dentro do contexto do nosso desenho de estudo, não é possível avaliar quanto os benefícios mostrados são específicos para pacientes infectados por COVID-19 versus os benefícios da anticoagulação na FA independentemente de COVID-19.

Ensaios randomizados anteriores avaliaram o impacto da anticoagulação em pacientes com COVID-19.^37-39^ Com base nesses estudos, tanto o Fórum de Anticoagulação quanto o Painel de Diretrizes de Tratamento para COVID-19 sugerem fornecer aos pacientes hospitalizados com infecção por SARS-CoV-2 uma dose profilática de heparina e uma dose terapêutica, por exemplo, em situações em que o paciente apresenta níveis elevados de dímero D e necessita de suporte de oxigenoterapia.^5,40^ A Sociedade Americana de Hematologia, porém, aconselha o uso de uma dose terapêutica em vez de uma dose profilática para pacientes hospitalizados com COVID-19.^41^

Mesmo assim, o regime e a dose ideais de anticoagulação para pacientes com COVID-19 permanecem controversos e seus efeitos sobre os resultados concretos são incertos.^40,41^ Por exemplo, ainda não foi determinado se a anticoagulação reduz a mortalidade em pacientes com COVID-19 internados em UTI.^42-44^ Além disso, o perfil de segurança dos ACOs nesta população de pacientes ainda não está claro, com dados observacionais sugerindo preocupações em relação às taxas de sangramento.^45^ Além disso, a enoxaparina terapêutica pode diminuir a necessidade de ventilação mecânica, embora isso não possa ser generalizado para anticoagulação oral no momento presente.^46^

Os pacientes que já estão em anticoagulação para condições subjacentes, como FA, podem apresentar menor risco tromboembólico ao desenvolverem infecção por COVID-19.^26^ Como não há consenso sobre quando exatamente o risco de tromboembolismo aumenta ao longo do curso da doença, aqueles que iniciam o tratamento com terapêutica anticoagulante após o diagnóstico de COVID-19 podem ainda ter uma janela de hipercoagulabilidade, com impacto pouco claro nos resultados.^47^ Isso pode ser particularmente importante em doentes com FA, que já apresentam um risco tromboembólico mais elevado devido à carga da doença e às comorbilidades.^48^ Nossa metanálise aborda esse assunto comparando a anticoagulação antes da aquisição da infecção por SARS-CoV-2 com ausência de anticoagulação prévia em pacientes com FA que posteriormente contraem COVID-19, indicando que a anticoagulação contínua pode afetar positivamente os desfechos de mortalidade de todas as causas e mortalidade relacionada à COVID-19.

A significância estatística de um desfecho pode ser afetada por vários fatores, por exemplo, tamanho da amostra, magnitude do efeito, variabilidade aleatória dos dados e o nível de confiança usado para testar a hipótese.^49^ Nesse caso, a mortalidade por todas as causas tem maior impacto clínico e significância estatística em comparação com a internação em UTI. Existem algumas explicações para esse efeito aparentemente discordante. Primeiro, os desfechos de mortalidade são menos propensos a vieses de medição.^50^ Em segundo lugar, a decisão de admitir um paciente na UTI é frequentemente influenciada por fatores individuais e locais, tais como disponibilidade de leitos, comorbidades do paciente, prognóstico geral e prioridade em relação a outros pacientes com doenças agudas.^51^

A maior heterogeneidade em nossos resultados merece discussão. Decidimos usar o modelo de efeitos aleatórios de MH porque previmos uma heterogeneidade considerável entre os estudos. Além disso, para abordar essa heterogeneidade, utilizamos 3 outros métodos: análise de sensibilidade de exclusão, gráfico de Baujat e diagnóstico de influência. Um estudo, Zadeh et al., destacou-se como um *outlier*.^31^ Este estudo incluiu apenas pacientes que apresentavam FA e insuficiência cardíaca. Dado o risco aumentado de eventos trombóticos em pacientes com insuficiência cardíaca, isso pode explicar o benefício exagerado do ACO neste estudo em relação a outros estudos de pacientes com FA, mas predominantemente sem insuficiência cardíaca.^52^

Esta metanálise apresenta algumas limitações. Primeiro, devido à natureza da comparação entre a presença versus ausência de anticoagulação crônica no momento do diagnóstico de COVID-19, apenas estudos observacionais puderam ser realizados e incluídos, o que pode introduzir viés de seleção inerente e fatores de confusão. No entanto, realizamos análises multivariadas ajustadas, quando possível, com resultados globalmente concordantes. É importante ressaltar que os pacientes em uso de ACO tiveram uma carga maior de comorbidades e ainda assim apresentaram redução da mortalidade por todas as causas e da mortalidade relacionada à COVID-19, o que aumenta a confiança nesses achados. Em segundo lugar, incluímos diferentes classes de ACO na análise conjunta, e não se sabe se existem efeitos diferenciais entre eles nesta população. Terceiro, a maioria dos estudos não mostrou nem comparou a dosagem de anticoagulantes tomados cronicamente, e as análises de sensibilidade que abordam esta limitação não puderam ser realizadas devido à falta de dados individuais ao nível do paciente. Além disso, por causa da ausência de dados específicos, não foi possível realizar análises para avaliar o desfecho de mortalidade para cada internação na UTI. Quarto, a heterogeneidade entre os estudos foi significativa nos principais resultados da nossa análise. Diferentes métodos foram utilizados para avaliar essa heterogeneidade e os resultados permaneceram consistentes nessas análises. Quinto, não foi possível avaliar resultados que pudessem determinar a gravidade da doença, como mortalidade para cada internação na UTI, devido a relatos incompletos nos resultados individuais e à ausência de dados no nível do paciente. Finalmente, não podemos atribuir os desfechos clínicos apenas ao uso prévio de terapia com ACO, uma vez que os pacientes frequentemente receberam terapias concomitantes antes e depois do diagnóstico de COVID-19. Esses fatores, sem dúvida, contribuíram para a heterogeneidade entre os estudos, limitando a nossa capacidade de analisar o efeito isolado dos anticoagulantes e de realizar metanálise de dados em subgrupos específicos.

Em relação aos pontos fortes, uma amostra considerável de mais de 1.177.000 pacientes foi incluída neste estudo. Além disso, nossa metanálise está relacionada a uma área essencial de pesquisa, pois aborda uma questão clínica significativa de anticoagulação crônica em pacientes com FA e infecção por COVID-19. Realizamos ainda análises ajustadas para avaliar os resultados controlando os fatores de confusão medidos, embora o risco de fatores de confusão residuais não possa ser excluído. No entanto, conforme apontado na Tabela 2, os pacientes em uso de ACO apresentaram maior carga de comorbidades e ainda apresentaram menor mortalidade por todas as causas e mortalidade relacionada à COVID-19, o que aumenta a confiança nesses achados. Em última análise, até onde sabemos, esta é a primeira metanálise que avalia os efeitos da anticoagulação crônica nesta população específica.

## Conclusões

Nesta metanálise de 10 estudos e 1.177.858 pacientes, a ACO crônica para FA em pacientes que posteriormente contraíram COVID-19 foi associada a taxas significativamente mais baixas de mortalidade por todas as causas e mortalidade por COVID-19 em comparação com nenhuma anticoagulação prévia.

## Material suplementar

Para informação adicional, por favor, clique aqui.
