# Supplementary material for: Anticoagulação Crônica em Pacientes com Fibrilação Atrial e COVID-19: Uma Revisão Sistemática e Metanálise
Source: Arq Bras Cardiol. 2024 Mar 21;121(3):e20230470. [Article in Portuguese] doi: 10.36660/abc.20230470 (PMC11081175; doi:10.36660/abc.20230470)
Supplement: Translation

# Translation: Chronic Anticoagulation in Patients with Atrial Fibrillation and COVID-19: A Systematic Review and Meta-Analysis

**DOI:** 10.36660/abc.20230470i

**Authors:** Isabela Landsteiner, Jonathan A. Pinheiro, Nicole Felix, Douglas Mesadri Gewehr, Rhanderson Cardoso

**Affiliations:** 1 Boston Massachusetts USA Massachusetts General Hospital, Boston, Massachusetts – USA; 2 Fortaleza CE Brazil Universidade de Fortaleza, Fortaleza, CE – Brazil; 3 Campina Grande PB Brazil Universidade Federal de Campina Grande, Campina Grande, PB – Brazil; 4 Curitiba PR Brazil Instituto do Coração de Curitiba, Curitiba, PR, Brazil; 5 Boston Massachusetts USA Brigham and Women's Hospital and Harvard Medical School, Boston, Massachusetts – USA

**Keywords:** Anticoagulants, Atrial Fibrillation, COVID-19, Factor Xa Inhibitors, Vitamin K

## Abstract

**Background:**

Coronavirus disease 2019 (COVID-19) is associated with hypercoagulability. It remains uncertain whether ongoing anticoagulation for atrial fibrillation (AF) in patients who later contract COVID-19 improves clinical outcomes.

**Objectives:**

To compare chronic oral anticoagulation with no previous anticoagulation in patients with AF who contracted a COVID-19 infection concerning the outcomes of all-cause mortality, COVID-19 mortality, intensive care unit (ICU) admission, and hospitalization.

**Methods:**

We systematically searched PubMed, Embase, and Cochrane Library for eligible studies from inception to December 2022. We included studies comparing COVID-19 outcomes in patients with versus without prior chronic anticoagulation for AF. Risk ratios (RR) with 95% confidence intervals (CI) were pooled with a random-effects model. The level of significance was set at p < 0.05. Quality assessment and risk of bias were performed according to Cochrane recommendations.

**Results:**

Ten studies comprising 1,177,858 patients with COVID-19 and AF were identified, of whom 893,772 (75.9%) were on prior chronic anticoagulation for AF. In patients with COVID-19, being on chronic anticoagulation for AF significantly reduced all-cause mortality (RR 0.75; 95% CI 0.57 to 0.99; p = 0.048; I^2^ = 89%) and COVID-19-related mortality (RR 0.76; 95% CI 0.72 to 0.79; p < 0.001; I^2^ = 0%) when compared with no prior anticoagulation. In contrast, there was no difference between groups regarding hospitalization (RR 1.08; 95% CI 0.82 to 1.41; p = 0.587; I^2^ = 95%) or ICU admission (RR 0.86; 95% CI 0.68 to 1.09; p = 0.216; I^2^ = 69%).

**Conclusions:**

In this meta-analysis, chronic anticoagulation for patients with AF who contracted COVID-19 was associated with significantly lower rates of all-cause mortality and COVID-19-related mortality as compared with no previous anticoagulation.

## Introduction

Coronavirus disease 2019 (COVID-19), caused by severe acute respiratory syndrome coronavirus-2 (SARS-CoV-2), emerged in December 2019, leading to a global pandemic with a high burden of morbidity, mortality, and economic hardship.^1^ The World Health Organization reported a total of 767 million confirmed cases and 6.9 million deaths worldwide as of July 5th, 2023.^2^

SARS-CoV-2 infection is associated with a wide myriad of non-respiratory clinical presentations, including pulmonary microvascular thrombosis and abnormal coagulation function, even in mild cases. The pathophysiology behind this refers mainly to immune-induced hypercoagulability secondary to the host's response to the infection.^3,4^ Taking this into consideration, guidelines have recommended that patients with COVID-19 on oral anticoagulation for underlying conditions and without any contraindications for it do not discontinue these interventions.^5^

Long-term therapy with oral anticoagulant (OAC) is an essential measure to avoid thromboembolic events in patients with atrial fibrillation (AF). The hypercoagulability associated with SARS-CoV-2 infection raises concerns about how ongoing anticoagulation in patients with AF who later contract COVID-19 can affect clinical outcomes.^5^ Therefore, we performed a systematic review and meta-analysis comparing the outcomes of anticoagulation versus no anticoagulation in patients with AF who developed COVID-19.

## Methods

This systematic review, meta-analysis, and reporting were performed following the Cochrane Collaboration Handbook for Systematic Reviews of Interventions and the Preferred Reporting Items for Systematic Reviews and Meta-Analysis (PRISMA) Statement guidelines.^6,7^ This meta-analysis was prospectively registered in the International Prospective Register of Systematic Reviews (PROSPERO) under the protocol number CRD42022341926.

### Search strategy and data extraction

We systematically searched PubMed, Embase, and the Cochrane Library from inception to December 2022. After removing duplicates, two authors (I.L. and N.F.) screened titles and abstracts and independently assessed full-text articles for inclusion based on prespecified criteria. Discrepancies were resolved through consensus between the authors. Additionally, we used backward snowballing (i.e., review of references) to identify relevant texts from articles identified in the original search.

Our search strategy included the following medical subject heading terms: "atrial fibrillation," "oral anticoagulant," "OACs," "NOAC," "non-vitamin K," "novel anticoagulant," "DOAC," "DOACs," "direct oral anticoagulant," "dabigatran," "apixaban," "edoxaban," "rivaroxaban," "VKA," "vitamin K antagonist," "warfarin," "LMWH," "low-molecular-weight heparin," "low molecular weight heparin," "enoxaparin," "bivalirudin," "dalteparin," "fondaparinux," "COVID-19," "coronavirus disease 19," "coronavirus disease-19," "SARS-CoV-2."

### Eligibility criteria

This systematic review and meta-analysis included studies that: (1) were full-length reports published in indexed journals or abstracts from major scientific conferences; (2) included adult patients (aged ≥ 18 years) previously diagnosed with AF who later contracted COVID-19 confirmed by a validated test; (3) stratified by patients on versus off chronic anticoagulation for AF; (4) reported of any of our outcomes of interest, namely all-cause mortality, COVID-19 mortality, intensive care unit (ICU) admission, and hospitalization; and (5) were published in English or Spanish. We excluded (1) studies with overlapping populations; (2) case reports, case series, letters to the editor, comments, or editorials; and (3) studies without control groups.

### Quality assessment

Observational studies were appraised using the Risk of Bias Summary for Non-randomized Studies (ROBINS-I) to assess the methodological quality of included studies, a tool based on answers to the signaling questions, judgments for each bias domain, and for overall risk of bias, which allows labeling each study as "low," "moderate," "serious," or "critical" risk of bias.^8^ Small study effect (publication bias) was assessed with funnel plots and Egger's regression test.^9^

### Statistical analysis

Binary endpoints were summarized using the Mantel-Haenszel (MH) random-effects model, with risk ratio (RR) and 95% confidence interval (CI) as a measure of effect size. We used the Sidik-Jonkman estimator (model error variance method) to calculate the heterogeneity variance τ ^2^, since the degree of heterogeneity was expected to be substantial.^10^

The adjusted hazard ratio (aHR) for multiple confounding factors, if reported, was pooled using the MH random-effects model with 95% CI and Paule-Mandel estimator for heterogeneity variance calculation. The heterogeneity among studies was evaluated with Cochrane's Q statistic (and the resulting chi-squared), with p ≤ 0.10 considered statistically significant. Higgins and Thompson's I^2^ statistic test was used to measure consistency.^11^ A value of 0% indicates no observed heterogeneity, and values of 1% to 25%, 26% to 50%, and > 50% indicate low, moderate, and substantial heterogeneity, respectively.^11^ Statistical significance was set at p < 0.05, and all tests were 2-tailed. We performed all calculations and graphics with R software version 4.2.2 (R Core Team 2021) using the extension packages "meta," "metafor," and "dmetar."^12-15^

### Addressing heterogeneity

We performed a graphic display of heterogeneity (GOSH) analysis for all-cause mortality to identify potential outliers and influential studies.^16^ First, a GOSH plot was generated. Then, three unsupervised machine learning (ML) algorithms were applied to detect clusters in the GOSH plot data, as follows: (1) the *k*-means algorithm;^17^ (2) density-based spatial clustering of applications with noise (DBSCAN);^18^ and (3) Gaussian mixture models.^19^

Alternatively, we applied "find.outliers" and "InfluenceAnalysis" functions (R dmetar package) for all-cause mortality, hospital admission, and ICU admission to assist in identifying potential outlier(s). The "find.outliers" function defines the study as an outlier if the study's confidence interval does not overlap with the confidence interval of the pooled effect. The "InfluenceAnalysis" function, in turn, generates three diagnostic plots: (1) the Baujat plot, used to identify studies that had high contributions to the heterogeneity in the meta-analytic data;^20^ (2) the leave-one-out sensitivity analysis by iteratively removing one study at a time to ensure the results were not dependent on a single study; and (3) influence diagnostics according to Viechbauer and Cheung.^21^

## Results

### Study selection and baseline characteristics

As illustrated in Figure 1, a total of 596 studies were identified, of which 493 were excluded based on title or abstract review. Twenty-six were fully reviewed based on our inclusion criteria. After the final appraisal, 10 manuscripts remained and were eligible for inclusion in this meta-analysis.^22-31^ A total of 1,177,858 patients were enrolled, of whom 893,772 (75.9%) were using anticoagulants for AF prior to a COVID-19 infection. The mean age ranged from 71 to 82 years, and 629,628 patients were male (55.5%). Individual study characteristics are reported in Table 1.

**Figure 1:** PRISMA flow diagram of study screening and selection. AF: atrial fibrillation; OAC: oral anticoagulants.

**Table 1:** Baseline characteristics of included studies

Study	Number of patients OAC / no OAC	Male, n (%)	Mean age, (years)	HTN, n (%)	HF, n (%)	Stroke, TIA, or systemic embolism, n (%)	Renal disease, n (%)	Liver disease, n (%)	PAD, n (%)	DM, n (%)	Lung disease, n (%)
Ageno, 2021^22^	43 / 111	91 (59.1)	81.0†	104 (67.5)	52 (33.8)	24 (15.6)	NA	NA	NA	30 (19.4)	43 (27.9)
Denas, 2021^23^	559 / 559	608 (54.4)	NA	972 (86.9)	184 (16.4)	169 (15.1)	96 (8.6)	11 (1.0)	27 (2.4)	298 (24.0)	NA
Flam, 2020^24^	103,703 / 36,875	87,508 (62.2)	73.6†	NA	30,986 (22.0)	28,803 (20.5)	7,823 (5.6)	2,227 (1.6)	NA	NA	27,855 (19.8)
Fumagalli, 2022^25^	91 / 85	91 (51.7)	82.0	125 (71.0)	55 (31.0)	23 (13.0)	36 (20.0)	3 (1.7)	37 (21.0)	58 (32.0)	37 (21.0)
Gómez, 2022^26^	1.361 / 392	1,065 (59.1)	78.1*	1,430 (79.4)	568 (31.5)	NA	NA	NA	174 (9.6)	555 (30.8)	269 (14.9)
Handy, 2022^27^	722,737 / 187,133	480,676 (52.8)	79.0	630,245 (69.2)	225,746 (24.8)	180,958 (19.9)	293,949 (32.3)	7,983 (0.9)	137,666 (15.1)	245,195 (26.9)	NA
Louis, 2022^28^	361 / 269	354 (56.1)	77.4	542 (86.0)	310 (49.2)	92 (14.6)	229 (36.3)	57 (9.0)	7 (1.1)	280 (44.4)	83 (13.2)
Rivera-Caravaca, 2022^29^	675 / 7.023	4,500 (58.4)	63.0†	3,718 (48.3)	174 (2.3)	570 (7.4)	484 (6.3)	271 (3.5)	645 (8.4)	1,455 (18.9)	523 (6.8)
Wong, 2022^30^	52,832 / 18,271	54,735 (76.9)	71.0†	29,683 (41.7)	7,124 (10.0)	2,244 (3.1)	10,524 (14.8)	NA	634 (0.9)	9,190 (12.9)	6,815 (9.6)
Zadeh, 2022^31^	11,410 / 33,368	NA	NA	NA	44,778 (100.0)	NA	NA	NA	NA	NA	NA

* Data regarding non-deceased patients.

† Data regarding anticoagulated patients. OAC: oral anticoagulant; HTN: hypertension; HF: heart failure; TIA: transient ischemic attack; PAD: peripheral artery disease; DM: diabetes mellitus

Given the non-randomized nature of the studies, we reported baseline characteristics stratified by use versus no use of anticoagulation among the 7 studies that reported such data (Table 2). As expected, there was a higher burden of comorbidities in patients with AF on OAC as compared with those not on OAC, including hypertension, heart failure, renal disease, and prior thromboembolic events. Patients on OAC were also older than those who were not anticoagulated.

**Table 2:** Individual study characteristics subdivided by anticoagulation use

Study	Number of patients OAC / no OAC	Male, n (%) OAC / no OAC	Mean age, (years) OAC / no OAC	HTN, n (%) OAC / no OAC	HF, n (%) OAC / no OAC	Stroke, TIA, or systemic embolism, n (%) OAC / no OAC	Renal disease, n (%) OAC / no OAC	Liver disease, n (%) OAC / no OAC	PAD, n (%) OAC / no OAC	DM, n (%) OAC / no OAC	Lung disease, n (%) OAC / no OAC
Ageno, 2021^22^	43 / 111	23 (53.5) / 68 (61.3)	81.0 / 80.0	30 (69.8) / 74 (66.7)	24 (55.8) / 28 (25.2)	8 (18.6) / 16 (14.4)	NA	NA	NA	10 (23.2) / 20 (18.0)	11 (25.6) / 32 (28.8)
Denas, 2021^23^	559 / 559	303 (54.2) / 306 (54.7)	NA	490 (87.7) / 485 (86.8)	93 (16.6) / 91 (16.3)	78 (14.0) / 91 (16.3)	47 (8.4) / 49 (8.8)	6 (1.1) / 5 (0.9)	15 (2.7) / 12 (2.1)	132 (23.6) / 137 (24.5)	NA
Flam, 2020^24^	103,703 / 36,875	62,488 (60.3) / 25,020 (67.9)	73.6 / 66.4	NA	26,544 (25.6) / 4,442 (12)	17,650 (17) / 2,853 (7.7)	6,082 (5.9) / 1,741 (4.7)	1,445 (1.4) / 832 (2.3)	NA	NA	21,762 (21) / 6,093 (16.5)
Handy, 2022^27^	722,737 / 187,133	384,260 (53.2) / 96,416 (10.6)	79.0 / 79.0	600,623 (83.1) / 124,731 (66.6)	228,877 (31.6) / 33,723 (18.0)	183,140 (25.3) / 30,370 (16.2)	284,379 (39.3) / 55,984 (29.9)	8,462 (1.17) / 2,543 (1.40)	159,892 (22.1) / 33,720 (18.0)	242,060 (33.5) / 47,979 (25.6)	NA
Louis, 2022^28^	361 / 269	206 (57.1) / 148 (55)	78.5 / 75.9	311 (86.1) / 231 (85.9)	200 (55.4) / 110 (40.9)	62 (17.2) / 30 (11.2)	113 (31.3) / 65 (24.2)	26 (7.2) / 31 (11.5)	4 (1.1) / 3 (1.1)	158 (43.8) / 122 (45.4)	55 (15.2) / 28 (10.4)
Rivera-Caravaca, 2022^29^	675 / 7,023	403 (59.7) / 4,097 (58.3)	80.0 / 63.0	543 (80.3) / 3,176 (45.2)	46 (6.8) / 128 (1.8)	131 (19.4) / 439 (6.3)	115 (17) / 369 (5.3)	33 (4.9) / 238 (3.4)	102 (15.1) / 543 (7.7)	198 (29.3) / 1,257 (17.9)	104 (15.4) / 419 (6.0)
Wong, 2022^30^	52,832 / 18,271	41,870 (79.2) / 12,865 (70.4)	71.0 / 69.0	22,061 (41.8) / 7,622 (41.7)	5,700 (10.8) / 1,424 (7.8)	1,041 (2.0) / 336 (1.8)	8,477 (16.0) / 2,047 (11.2)	NA	426 (0.8) / 208 (1.1)	6,508 (12.3) / 2,682 (14.6)	7,372 (14) / 2,302 (12.6)

OAC: oral anticoagulant; HTN: hypertension; HF: heart failure; TIA: transient ischemic attack; PAD: peripheral artery disease; DM: diabetes mellitus

### Pooled analysis of all studies

Mortality endpoints are summarized in Figure 2A-D. All-cause mortality (Figure 2A) and COVID-19-related mortality (Figure 2B) were significantly reduced among patients receiving OAC therapy compared with those without prior use of OAC. We conducted a prespecified subgroup analysis by pooling studies that reported aHR for COVID-19 mortality while taking multiple confounding factors into account. In this sensitivity analysis, OAC therapy remained significantly associated with reduced COVID-19-related mortality (Figure 2C). When stratified by the type of OAC therapy, there was no significant difference between non-vitamin K antagonist oral anticoagulant (NOAC) therapy versus vitamin K antagonist (VKA) therapy for all-cause mortality (Figure 2D).

**Figure 2:** Meta-analysis of mortality outcomes in patients with atrial fibrillation undergoing OAC therapy. Forest plots presenting the risk ratio (RR), or hazard ratio (HR), and 95% confidence interval (CI) on (A) all-cause mortality, (B) COVID-19-related mortality, (C) COVID-19-related mortality (adjusted HR), and (D) all-cause mortality NOAC versus VKA. CI: confidence interval; HR: hazard ratio; MH: Mantel-Haenszel; NOAC: non-vitamin K oral antagonist; OAC: oral anticoagulants; RR: risk ratio; VKA: vitamin K antagonist.

The incidences of hospital admission (Figure 3A) and ICU admission (Figure 3B) were similar between OAC versus non-OAC therapy, with substantial between-study heterogeneity in both outcomes. Considering this, we performed a sensitivity analysis for the endpoint of hospital admission by including only studies that reported aHR by multivariable models or propensity score matching, which retrieved results consistent with the overall analysis and eliminated between-study heterogeneity (Figure 3C).

**Figure 3:** Meta-analysis of hospital outcomes in patients with atrial fibrillation undergoing OAC therapy. Forest plots presenting the risk ratio (RR), or hazard ratio (HR), and 95% confidence interval (CI) on (A) hospital admission, (B) intensive care unit admission, and (C) hospital admission (adjusted HR). CI: confidence interval; HR: hazard ratio; MH: Mantel-Haenszel; OAC: oral anticoagulants; RR: risk ratio.

### Addressing heterogeneity

Considering the significant between-study heterogeneity retrieved in key outcomes, we performed GOSH analyses for all-cause mortality, our primary endpoint. The GOSH plot illustrates the effect size plotted against the I^2^ for all possible combinations of studies. The 255 possible subsets of meta-analysis (2*κ* – 1 possible combinations) for all-cause mortality are presented as a GOSH plot in Figure 4A. By analyzing the pattern in our data, we find that most values are concentrated in a cluster with high heterogeneity. The distribution of I^2^ is relatively unimodal, although there seem to be some study combinations sparsely distributed for which the estimated heterogeneity is slightly lower, with a pooled effect size reasonably preserved, resulting in an overall I^2^ distribution skewed down-leftwards.

**Figure 4:** Addressing Heterogeneity. (4A) GOSH Plot; (4B) K-means algorithm; (4C) DBSCAN algorithm; (4D) Gaussian mixture model; (4E) GOSH plot with the corresponding subset (Zadeh et al., 2022), including the potential outliers colored in cyan; (4F) Leave-one-out analysis; (4G) Baujat Plot; (4H) Influence Diagnostics. CI: confidence interval; DBSCAN: density-based spatial clustering of applications with noise; GMM: Gaussian mixture model; GOSH: graphic display of heterogeneity; OAC: oral anticoagulants; RR: risk ratio.

To identify which studies may have caused this shape, we applied three unsupervised ML algorithms, detailed in methods, to detect clusters in the GOSH plot data (Figures 4B-D). Ultimately, one potential outlier was identified by these ML tools.^31^ The corresponding subset, including our potential outlier, is demonstrated in Figure 4E. In summary, the GOSH analysis showed that heterogeneity did not significantly change, no matter which publication was excluded, nor did the overall effect. This is most consistent with the interpretation that our results are robust and reliable, although the overall between-study heterogeneity may be significant.

Because the GOSH analysis remained heterogeneous for all-cause mortality, we further explored the influence of each study by performing a leave-one-out sensitivity analysis (Figure 4F), plotting the Baujat plot (Figure 4G), and performing influence diagnostics (Figure 4H). Leave-one-out analysis and Baujat plot showed that Zadeh et al. had the highest contribution to high heterogeneity, consistent with the results of the GOSH analysis.^31^ Furthermore, the pooled effect estimates (RR) in the leave-one-out analysis ranged from 0.70 to 0.85. By excluding Flam et al.,^24^ Gómez et al.,^26^ and Rivera-Caravaca et al.,^29^ the resultant effect size remained statistically significant. In tandem, we applied the InfluenceAnalysis function (R dmetar package) to verify if another influential case recognition approach detected the same outliers found in the aforementioned analyses. Influential diagnostics characterized which studies fit well into our meta-analysis model and which did not.

### Quality assessment

The ROBINS-I found 7 studies at a moderate overall risk of bias, while 3 were identified as having a serious overall risk of bias (Supplementary Table S1). Funnel plots for all-cause mortality, hospital admission, and ICU admission were slightly asymmetrical (Supplementary Table S2A-C). However, Egger's test for publication bias was only statistically significant for the outcome of ICU admission (p = 0.04) (Supplementary Table S2C).

## Discussion

In this systematic review and meta-analysis of 10 observational studies and 1,177,858 patients that compared chronic oral anticoagulation with no previous anticoagulation in patients with AF who contracted COVID-19, our main findings were as follows: (1) all-cause mortality and COVID-19-related mortality were significantly lower in patients on chronic OAC therapy; and (2) the association of OAC therapy with a reduction in COVID-19-related mortality persisted even after a pooled analysis of hazard ratios adjusted for multiple confounding factors.

The association between AF and adverse outcomes is well documented in the literature, as AF significantly increases the risk of stroke, systemic embolism, and mortality.^32-35^ In this context, oral anticoagulation substantially improves cardiovascular endpoints and, ultimately, survival in patients with AF.^32-34^ Specifically, oral anticoagulation has been shown to reduce the risk of ischemic stroke by 64% and all-cause mortality by 26%.^34^ Since AF is associated with worse outcomes in patients with COVID-19, the benefit of anticoagulation may be even greater in patients with AF who are infected with COVID-19.^36^ How much of the benefits shown in our study are particular to COVID-19 infected patients versus the benefits of anticoagulation in AF regardless of COVID-19 cannot be evaluated in the context of our study design.

Previous randomized trials have assessed the impact of anticoagulation on patients with COVID-19.^37-39^ Based on these studies, both the Anticoagulation Forum and the COVID-19 Treatment Guidelines Panel suggest providing hospitalized patients with SARS-CoV-2 infection a prophylactic dose of heparin and a therapeutic dose, for instance in situations when the patient has high levels of D-dimer and needs oxygen therapy support.^5,40^ The American Society of Hematology, however, advises using a therapeutic dose over a prophylactic one for hospitalized patients with COVID-19.^41^

Even so, the optimal anticoagulation regimen and dose for patients with COVID-19 remains controversial, and its effects on hard outcomes are uncertain.^40,41^ For instance, whether anticoagulation reduces mortality in patients with COVID-19 admitted to the ICU is yet to be determined.^42-44^ In addition, the safety profile of OACs in this patient population is still unclear, with observational data suggesting concerns regarding bleeding rates.^45^ Moreover, therapeutic enoxaparin may decrease the need for mechanical ventilation, even though this cannot be generalized to oral anticoagulation at present.^46^

Patients already on anticoagulation for underlying conditions such as AF may be at a lower thromboembolic risk when they develop COVID-19 infection.^26^ Since there is no consensus about when exactly the risk of thromboembolism increases throughout the course of the disease, those who start the anticoagulation therapy after being diagnosed with COVID-19 may still have a window of hypercoagulability, with unclear impact on outcomes.^47^ This may be particularly important in patients with AF, who are already at a higher thromboembolic risk due to disease burden and comorbidities.^48^ Our meta-analysis addresses this matter by comparing anticoagulation prior to the acquisition of the SARS-CoV-2 infection with no previous anticoagulation in patients with AF who later contract COVID-19, indicating that ongoing anticoagulation may positively affect the outcomes of all-cause mortality and COVID-19-related mortality.

The statistical significance of an outcome may be affected by several factors, such as sample size, magnitude of effect, random variability of data, and confidence level used to test the hypothesis.^49^ In this instance, all-cause mortality has a greater clinical and statistical significance compared with ICU admission. There are a couple of explanations for this apparently discordant effect. First, mortality outcomes are less prone to measurement bias.^50^ Second, the decision to admit a patient to the ICU is often influenced by individual and local factors, such as bed availability, patient comorbidities, overall prognosis, and priority relative to other acutely ill patients.^51^

The increased heterogeneity in our results warrants discussion. We decided to use the MH random-effects model because we anticipated considerable between-study heterogeneity. In addition, in order to address this heterogeneity, we used three other methods: leave-one-out sensitivity analysis, Baujat plot, and influence diagnostics. One study, Zadeh et al., stood out as an outlier.^31^ This study only included patients who had both AF and heart failure. Given the increased risk of thrombotic events in patients with heart failure, this may explain the exaggerated benefit of OAC in this study relative to other studies of patients with AF, but predominantly without heart failure.^52^

This meta-analysis has some limitations. First, due to the nature of the comparison between the presence versus absence of chronic anticoagulation at the time of COVID-19 diagnosis, only observational studies could be performed and included, which may introduce inherent selection bias and confounders. Nevertheless, we performed multivariable adjusted analyses, when possible, with overall concordant results. Importantly, patients on OAC had a higher burden of comorbidities and yet were still found to have reduced all-cause mortality and COVID-19 related mortality, which increases the confidence in these findings. Second, we included different classes of OAC in the pooled analysis, and it is unknown if there are any differential effects between them in this population. Third, most studies neither showed nor compared the dosage of anticoagulants taken chronically, and sensitivity analyses addressing this limitation could not be performed due to a lack of individual patient-level data. Additionally, due to this lack of specific data, analysis to assess the outcome of mortality per ICU admission could not be performed. Fourth, between-study heterogeneity among studies was significant in the key outcomes of our analysis. Different methods were performed to evaluate this heterogeneity, and the results remained consistent in those analyses. Fifth, we were unable to evaluate outcomes that could ascertain disease severity such as mortality per ICU admission due to incomplete reporting in the individual results and absence of patient-level data. Finally, we cannot attribute clinical outcomes solely to the previous use of OAC therapy, given that patients often received concomitant therapies both pre- and post-COVID-19 diagnosis. These factors undoubtedly contributed to the heterogeneity among studies, limiting our ability to analyze the isolated effect of anticoagulants and meta-analyze data on specific subgroups.

Regarding the strengths, a considerable sample size of over 1,177,000 patients was included in this study. In addition, our meta-analysis is related to an essential area of research as it addresses a significant clinical question of chronic anticoagulation in patients with AF and a COVID-19 infection. Furthermore, we performed adjusted analyses to evaluate outcomes controlling for measured confounders, although the risk of residual confounders cannot be excluded. Nevertheless, as pointed out in Table 2, patients on OAC had a higher burden of comorbidities and were still found to have lower all-cause mortality and COVID-19-related mortality, which increases the confidence in these findings. Ultimately, to our best knowledge, this is the first meta-analysis evaluating the effects of chronic anticoagulation in this specific population.

## Conclusions

In this meta-analysis of 10 studies and 1,177,858 patients, chronic OAC for AF in patients who later contracted COVID-19 was associated with significantly lower rates of all-cause mortality and mortality due to COVID-19 compared with no previous anticoagulation.

## Supplemental Materials

For additional information, please click here.
